# Ureteral stone with hydronephrosis and urolithiasis alone are risk factors for acute kidney injury in patients with urinary tract infection

**DOI:** 10.1038/s41598-021-02647-8

**Published:** 2021-12-02

**Authors:** Chih-Yen Hsiao, Tsung-Hsien Chen, Yi-Chien Lee, Ming-Cheng Wang

**Affiliations:** 1 Division of Nephrology, Department of Internal Medicine, Ditmanson Medical Foundation Chia-Yi Christian Hospital, Chia-Yi, Taiwan; 2 Department of Hospital and Health Care Administration, Chia Nan University of Pharmacy and Science, Tainan, Taiwan; 3 Department of Internal Medicine, Ditmanson Medical Foundation Chia-Yi Christian Hospital, Chia-Yi, Taiwan; 4 Department of Internal Medicine, Fu Jen Catholic University Hospital, Fu Jen Catholic University, New Taipei, Taiwan; 5 School of Medicine, College of Medicine, Fu Jen Catholic University, New Taipei, Taiwan; 6 Division of Nephrology, Department of Internal Medicine, National Cheng Kung University Hospital, College of Medicine, National Cheng Kung University, Tainan, Taiwan

**Keywords:** Medical research, Nephrology, Urology

## Abstract

To identify whether urolithiasis with or without hydronephrosis has an impact on acute kidney injury (AKI) in patients with urinary tract infection (UTI). This study aimed to identify whether urolithiasis with or without hydronephrosis has an impact on AKI in patients with UTI. This retrospective study enrolled hospitalized UTI patients who underwent imaging in an acute care setting from January 2006 to April 2019. Of the 1113 participants enrolled, 191 (17.2%) had urolithiasis and 76 (6.8%) had ureteral stone complicated with hydronephrosis. Multivariate logistic regression analysis showed that in UTI patients with urolithiasis, the presence of ureteral stone with concomitant hydronephrosis was an independent risk factor for AKI (odds ratio [OR] 2.299, 95% confidence interval [CI] 1.112–4.755, P = 0.025). In addition, urolithiasis was associated with an increased risk for AKI (OR 2.451, 95% CI 1.369–4.389, P = 0.003) in UTI patients without hydronephrosis. The presence of ureteral stone with hydronephrosis increases the risk for AKI of UTI patients with urolithiasis, and urolithiasis remains a risk factor of AKI in UTI patients without hydronephrosis.

## Introduction

Urolithiasis is a widespread problem with a male preponderance, showing a lifetime prevalence of 10% in men and 5% in women^1,2^. The worldwide prevalence of urolithiasis is also steadily increasing^1–3^, partially owing to its strong association with metabolic syndrome conditions such as obesity, hypertension, and diabetes^4^.

Urolithiasis can cause secondary complications, such as obstruction or urinary tract infection (UTI)^5^. Acute UTI may cause sudden deterioration of renal function, especially in the presence of urinary tract obstruction^6^. Our previous study showed that urolithiasis is a risk factor for acute kidney injury (AKI) among patients with UTI^7^, and AKI has been associated with short- and long-term mortality^8^. Since AKI leads to poor outcomes, recognizing the risk factors for its development in patients with urolithiasis and UTI is crucial. However, no study has focused on whether urolithiasis without hydronephrosis is a risk factor for the development of AKI in patients with UTI, or whether hydronephrosis caused by ureteral stone is a risk factor for the development of AKI in UTI patients with urolithiasis. Hence, we conducted this study to investigate these important issues.

## Results

### Characteristics of the study population

**Figure 1:**
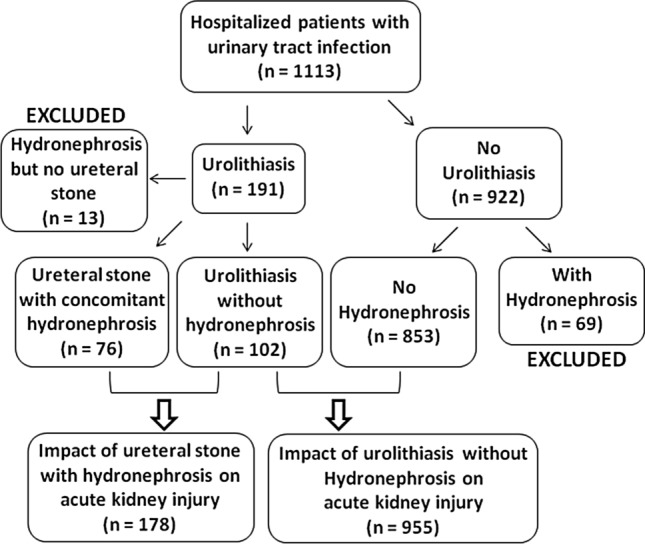
Flowchart of the selection of study subjects.

**Figure 2:**
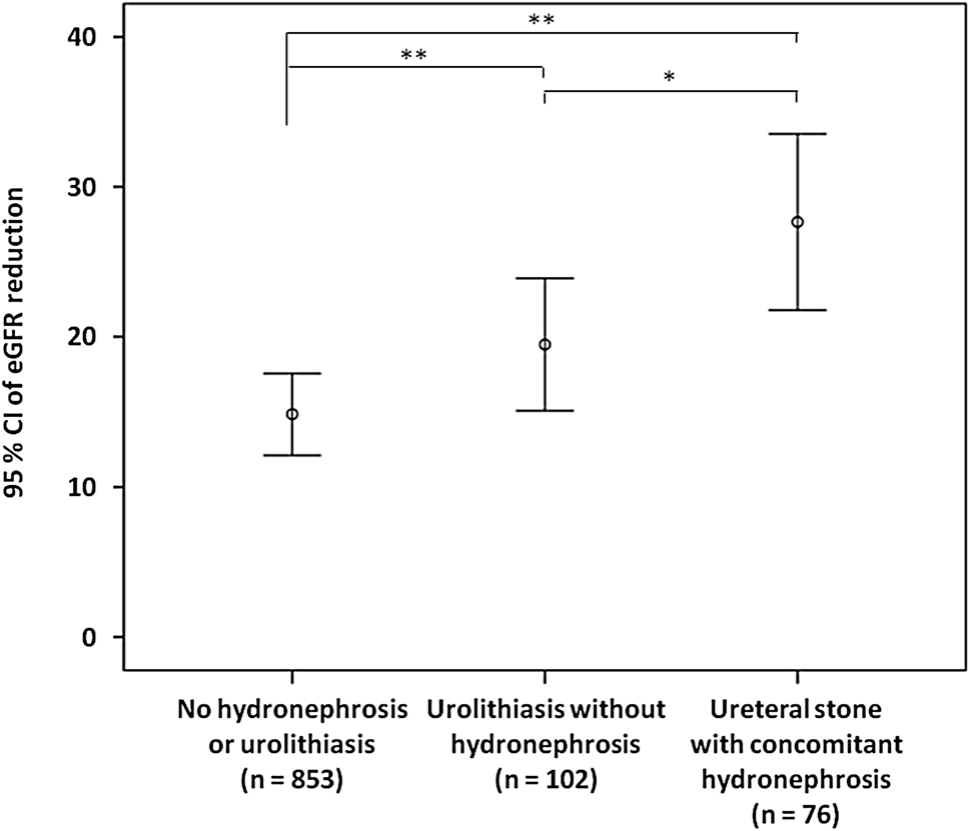
eGFR reduction in UTI patients with respect to ureteral stone with concomitant hydronephrosis and urolithiasis without hydronephrosis. *eGFR* estimated glomerular filtration rate, *UTI* urinary tract infection, *eGFR reduction* baseline minus worst value of eGFR. *P < 0.05, **P < 0.01.

The demographic and clinical characteristics of the 1113 hospitalized UTI patients included in this study are shown in Table 1. The included patients were divided into three groups: UTI patients with no hydronephrosis or urolithiasis, UTI patients with ureteral stone and concomitant hydronephrosis, or UTI patients with urolithiasis but no hydronephrosis (Fig. 1). The mean age on admission was 66 ± 17 years. Most of the patients (816 [73.3%]) were women, and 375 (33.7%) had a history of prior UTI. Of the patients, 220 (19.8%) had uroseptic shock and 165 (14.8%) had AKI during hospitalization. The overall mortality rate was 0.6% (7/1113). Among UTI patients, 191 (17.2%) had urolithiasis and 76 (6.8%) had ureteral stone with hydronephrosis. Among 76 UTI patients with ureteral stone and hydronephrosis, five (6.6%) had bilateral ureteral stones complicated with bilateral hydronephrosis. Figure 2 shows a reduction in estimated glomerular filtration rate (eGFR) in different groups of hospitalized patients with UTI. The reduction in eGFR in UTI patients without urolithiasis or hydronephrosis, in those with urolithiasis but without hydronephrosis, and in those with ureteral stone and concomitant hydronephrosis was 13.45 (95% confidence interval [CI] 12.44–14.45), 20.54 (95% CI 16.34–24.73), and 27.65 (95% CI 21.77–33.53) mL/min/1.73 m^2^, respectively. Thirteen urolithiasis patients who had hydronephrosis but with no ureteral stone identified in imaging studies and 69 patients who did not have urolithiasis but had hydronephrosis were excluded from further analysis (Fig. 1). The use of antibiotics was not included in subsequent univariate and multivariate logistic regression analyses because there is no statistical significance in multivariate analysis or after excluding those developing AKI before antibiotic administration.

**Table 1:** Characteristics of hospitalized patients with urinary tract infection with respect to urolithiasis.

Characteristic	All (n = 1113)	Urolithiasis		*P*-value
		Non (n = 922)	Yes (n = 191)	
Age (year)	66 ± 17	66 ± 18	67 ± 14	0.413^##^
Gender (male)	297 (26.7)	231 (25.1)	66 (34.6)	0.007^¥^
Diabetes mellitus	478 (42.9)	391 (42.4)	87 (45.5)	0.425^¥^
Hypertension	584 (52.5)	476 (51.6)	108 (56.5)	0.215^¥^
Congestive heart failure	47 (4.2)	38 (4.1)	9 (4.7)	0.712^¥^
Coronary artery disease	122 (11.0)	100 (10.8)	22 (11.5)	0.788^¥^
Stroke	237 (21.3)	198 (21.5)	39 (20.4)	0.746^¥^
Prior history of UTI				0.091^¥^
None	738 (66.3)	622 (67.5)	116 (60.7)	
Once	212 (19.0)	174 (18.9)	38 (19.9)	
Twice	89 (8.0)	72 (7.8)	17 (8.9)	
Thrice or more	74 (6.6)	54 (5.9)	20 (10.5)	
Indwelling Foley catheter	70 (6.3)	56 (6.1)	14 (7.3)	0.515^¥^
Afebrile	405 (36.4)	342 (37.1)	63 (33.0)	0.283^¥^
Bacteremia	519 (46.6)	408 (44.3)	111 (58.1)	< 0.001^¥^
Uroseptic shock	220 (19.8)	163 (17.7)	57 (29.8)	< 0.001^¥^
Acute kidney injury	165 (14.8)	105 (11.4)	60 (31.4)	< 0.001^¥^
Length of hospital stay (day)	9 ± 5	9 ± 5	11 ± 5	< 0.001^#^
All-cause in-hospital mortality	7 (0.6)	6 (0.7)	1 (0.5)	1.000^¥^
Hospitalized serum creatinine (mg/dL)	1.59 ± 1.60	1.52 ± 1.52	1.93 ± 1.88	< 0.001^##^
Baseline eGFR (mL/min/1.73 m^2^)	76.12 ± 30.27	76.57 ± 30.80	73.97 ± 27.58	0.281^#^
BUN/creatinine ratio > 20	356 (32.0)	303 (32.9)	53 (27.7)	0.168^¥^
Mean white blood cell (10^3^/μL)	13.31 ± 6.22	13.21 ± 6.03	13.82 ± 7.07	0.212^#^
Platelets (10^3^/μL)	204.64 ± 111.06	206.99 ± 116.05	193.3 ± 82.16	0.121^#^
*Escherichia coli*	876 (78.7)	747 (81.0)	129 (67.5)	< 0.001^¥^
*Proteus* species	34 (3.1)	16 (1.7)	18 (9.4)	< 0.001^¥^
*Klebsiella* species	78 (7.0)	63 (6.8)	15 (7.9)	0.615^¥^
*Enterococcus* species	39 (3.5)	32 (3.5)	7 (3.7)	0.894^¥^
*Pseudomonas* species	55 (4.9)	45 (4.9)	10 (5.2)	0.837^¥^
Nephrotoxic agents	664 (59.9)	547 (59.7)	117 (61.3)	0.680^¥^
Cephalosporins	748 (67.2)	626 (67.9)	122 (63.9)	0.281^¥^
Fluoroquinolones	110 (9.9)	89 (9.7)	21 (11.0)	0.572^¥^
Penicillins	133 (11.9)	107 (11.6)	26 (13.6)	0.436^¥^
Aminoglycosides	80 (7.2)	69 (7.5)	11 (5.8)	0.401^¥^
Carbapenems	80 (7.2)	62 (6.7)	18 (9.4)	0.189^¥^
Multidrug-resistance bacterial isolate	390 (35.0)	318 (34.5)	72 (37.7)	0.398^¥^

*UTI* urinary tract infection, *eGFR* estimated glomerular filtration rate, *BUN* blood urea nitrogen.

^#^Student T-test.

^##^Mann–Whitney *U*-test.

^¥^Chi-Square test or Fisher’s exact test.

### Risk factors of hospitalized UTI patients with urolithiasis with respect to ureteral stone and concomitant hydronephrosis

The demographic and clinical characteristics of UTI patients with urolithiasis are shown in Table 2. Patients with ureteral stone and concomitant hydronephrosis had higher serum creatinine level on admission (2.35 ± 2.28 versus 1.58 ± 1.41 mg/mL, P = 0.02), longer hospital stay (12 ± 6 versus 9 ± 4 days, P < 0.001), higher prevalence of bacteremia (69.7% versus 49.0%, P = 0.006), AKI (40.8% versus 22.5%, P = 0.009) and the use of carbapenems (18.4% versus 2.9%, P = 0. 001), and lower prevalence of afebrile status (19.7% versus 40.2%, P = 0.004) and the use of cephalosporines (56.6% versus 72.5%, P = 0. 026) than those without ureteral stone and concomitant hydronephrosis. Univariate logistic regression analysis showed that older age (odds ratio [OR] 1.032, 95% CI 1.006–1.057, P = 0.014), uroseptic shock (OR 6.249, 95% CI 3.074–12.702, P < 0.001), and ureteral stone with concomitant hydronephrosis (OR 2.366, 95% CI 1.233–4.541, P = 0.01) were associated with an increased risk for AKI, whereas a higher platelet count (OR 0.995, 95% CI 0.991–1.000, P = 0.042) was associated with a decreased risk for AKI in UTI patients with urolithiasis. Multivariate logistic regression analysis revealed that older age (OR 1.030, 95% CI 1.000–1.060, P = 0.047), uroseptic shock (OR 5.605, 95% CI 2.683–11.709, P < 0.001), and ureteral stone with concomitant hydronephrosis (OR 2.299, 95% CI 1.112–4.755, P = 0.025) were independently associated with an increased risk for AKI in UTI patients with urolithiasis (Table 3).

**Table 2:** Characteristics of hospitalized urinary tract infection patients with urolithiasis with respect to ureteral stone with hydronephrosis.

Characteristic	All (n = 178)	Ureteral stone with hydronephrosis		*P*-value
		Non (n = 102)	Yes (n = 76)	
Age (year)	67 ± 14	66 ± 16	67 ± 11	0.717^##^
Gender (male)	60 (33.7)	39 (38.2)	21 (27.6)	0.139^¥^
Diabetes mellitus	81 (45.5)	48 (47.1)	33 (43.4)	0.630^¥^
Hypertension	101 (56.7)	60 (58.8)	41 (53.9)	0.516^¥^
Congestive heart failure	8 (4.5)	5 (4.9)	3 (3.9)	1.000^¥^
Coronary artery disease	20 (11.2)	11 (10.8)	9 (11.8)	0.825^¥^
Stroke	37 (20.8)	18 (17.6)	19 (25)	0.232^¥^
Prior history of UTI				0.638^¥^
None	104 (58.4)	56 (54.9)	48 (63.2)	
Once	38 (21.3)	25 (24.5)	13 (17.1)	
Twice	16 (9.0)	9 (8.8)	7 (9.2)	
Thrice or more	20 (11.2)	12 (11.8)	8 (10.5)	
Indwelling Foley catheter	13 (7.3)	8 (7.8)	5 (6.6)	0.748^¥^
Afebrile	56 (31.5)	41 (40.2)	15 (19.7)	0.004^¥^
Bacteremia	103 (57.9)	50 (49.0)	53 (69.7)	0.006^¥^
Uroseptic shock	53 (29.8)	26 (25.5)	27 (35.5)	0.148^¥^
Acute kidney injury	54 (30.3)	23 (22.5)	31 (40.8)	0.009^¥^
Length of hospital stay (day)	11 ± 5	9 ± 4	12 ± 6	< 0.001^##^
All-cause in-hospital mortality	1 (0.6)	0 (0.0)	1 (1.3)	0.427^¥^
Hospitalized serum creatinine (mg/dL)	1.91 ± 1.86	1.58 ± 1.41	2.35 ± 2.28	0.020^##^
Baseline eGFR (mL/min/1.73 m^2^)	74.24 ± 27.59	76.36 ± 26.61	71.40 ± 28.78	0.237^#^
BUN/creatinine ratio > 20	51 (28.7)	32 (31.4)	19 (25.0)	0.352^¥^
Mean white blood cell (10^3^/μL)	13.94 ± 7.14	13.82 ± 6.24	14.09 ± 8.23	0.803^#^
Platelets (10^3^/μL)	196.31 ± 80.96	200.12 ± 84.17	191.21 ± 76.70	0.469^#^
*Escherichia coli*	124 (69.7)	74 (72.5)	50 (65.8)	0.332^¥^
*Proteus* species	17 (9.6)	8 (7.8)	9 (11.8)	0.369^¥^
*Klebsiella* species	14 (7.9)	7 (6.9)	7 (9.2)	0.565^¥^
*Enterococcus* species	6 (3.4)	2 (2.0)	4 (5.3)	0.404^¥^
*Pseudomonas* species	8 (4.5)	5 (4.9)	3 (3.9)	1.000^¥^
Nephrotoxic agents	110 (61.8)	57 (55.9)	53 (69.7)	0.060^¥^
Cephalosporins	117 (65.7)	74 (72.5)	43 (56.6)	0.026^¥^
Fluoroquinolones	19 (10.7)	12 (11.8)	7 (9.2)	0.585^¥^
Penicillins	24 (13.5)	13 (12.7)	11 (14.5)	0.738^¥^
Aminoglycosides	11 (6.2)	6 (5.9)	5 (6.6)	1.000^¥^
Carbapenems	17 (9.6)	3 (2.9)	14 (18.4)	0.001^¥^
Multidrug-resistance bacterial isolate	65 (36.5)	37 (36.3)	28 (36.8)	0.938^¥^

*UTI* urinary tract infection, *eGFR* estimated glomerular filtration rate, *BUN* blood urea nitrogen.

Data are expressed as mean ± SD or number (percentage) ^#^Student T-test; ^##^Mann–Whitney U-test; ^¥^Chi-Square test or Fisher’s exact test.

**Table 3:** Univariate and multivariate logistic regression analyses of factors related to acute kidney injury in hospitalized urinary tract infection patients with urolithiasis.

Covariate	Univariate			Multivariate		
	*β*	OR (95% CI)	*P*-value	*β*	OR (95% CI)	*P*-value
Age (year)	0.031	1.032 (1.006–1.057)	0.014	0.029	1.030 (1.000–1.060)	0.047
Gender (male)	− 0.144	0.865 (0.437–1.714)	0.679			
Diabetes mellitus	0.046	1.047 (0.551–1.988)	0.889			
Hypertension	0.148	1.159 (0.606–2.217)	0.655			
Congestive heart failure	0.875	2.400 (0.577–9.975)	0.228			
Coronary artery disease	− 0.018	0.982 (0.356–2.709)	0.972			
Stroke	0.430	1.537 (0.720–3.282)	0.267			
Indwelling Foley catheter	0.022	1.022 (0.301–3.475)	0.972			
Afebrile	0.363	1.438 (0.732–2.824)	0.292			
Bacteremia	0.417	1.517 (0.783–2.938)	0.217			
Uroseptic shock	1.832	6.249 (3.074–12.702)	< 0.001	1.724	5.605 (2.683–11.709)	< 0.001
Ureteral stone with hydronephrosis	0.861	2.366 (1.233–4.541)	0.010	0.833	2.299 (1.112–4.755)	0.025
Baseline eGFR (mL/min/1.73 m^2^)	− 0.001	0.999 (0.988–1.011)	0.877			
BUN/creatinine ratio > 20	0.445	1.561 (0.784–3.106)	0.205			
Mean white blood cell (10^3^/μL)	0.043	1.044 (0.998–1.092)	0.062			
Platelets (10^3^/μL)	− 0.005	0.995 (0.991–1.000)	0.042	− 0.003	0.997 (0.992–1.001)	0.135
Nephrotoxic agents	− 0.485	0.616 (0.321–1.180)	0.144			
Multidrug-resistance bacterial isolate	0.482	1.620 (0.842–3.116)	0.149			

Cox & Snell R^2^ = 0.197, Nagelkerke R^2^ = 0.278.

*eGFR* estimated glomerular filtration rate, *BUN* blood urea nitrogen.

### Risk factors of hospitalized UTI patients without hydronephrosis with respect to urolithiasis

The demographic and clinical characteristics of UTI patients without hydronephrosis are shown in Table 4. Patients with urolithiasis had a higher prevalence of male gender (38.2% versus 25.3%, P = 0.005), prior history of UTI (45.1% versus 31.4%, P = 0.016), AKI (22.5% versus 9.6%, P < 0.001), and *Proteus* species (7.8% versus 1.6%, P = 0.001), but a lower prevalence of *Escherichia coli *(*E. coli*) (72.5% versus 80.9%, P = 0.047), than those without urolithiasis. Univariate logistic regression analysis showed that older age (OR 1.029, 95% CI 1.015–1.043, P < 0.001), diabetes mellitus (DM) (OR 1.549, 95% CI 1.031–2.326, P = 0.035), hypertension (OR 2.596, 95% CI 1.665–4.048, P < 0.001), congestive heart failure (CHF) (OR 2.691, 95% CI 1.282–5.646, P = 0.009), afebrile status (OR 1.556, 95% CI 1.034–2.340, P = 0.034), bacteremia (OR 1.696, 95% CI 1.128–2.550, P = 0.011), uroseptic shock (OR 4.318, 95% CI 2.814–6.627, P < 0.001), urolithiasis (OR 2.737, 95% CI 1.632–4.591, P < 0.001), and higher white blood cell (WBC) count (OR 1.059, 95% CI 1.027–1.092, P < 0.001) were associated with an increased risk for AKI, whereas a higher baseline eGFR (OR 0.982, 95% CI 0.976–0.989, P < 0.001) and the use of nephrotoxic agents (OR 0.447, 95% CI 0.296–0.675, P < 0.001) were associated with a decreased risk for AKI in UTI patients without hydronephrosis. Multivariate logistic regression analysis revealed that hypertension (OR 2.125, 95% CI 1.255–3.599, P = 0.005), uroseptic shock (OR 4.683, 95% CI 2.880–7.613, P < 0.001), urolithiasis (OR 2.451, 95% CI 1.369–4.389, P = 0.003), and higher WBC count (OR 1.069, 95% CI 1.034–1.105, P < 0.001) were independently associated with an increased risk for AKI in UTI patients without hydronephrosis. Conversely, a higher baseline eGFR (OR 0.987, 95% CI 0.979–0.996, P = 0.004) and the use of nephrotoxic agents (OR 0.510, 95% CI 0.319–0.816, P = 0.005) were independently associated with a decreased risk for AKI in UTI patients without hydronephrosis (Table 5).

**Table 4:** Characteristics of hospitalized urinary tract infection patients without hydronephrosis with respect to urolithiasis.

Characteristic	All (n = 955)	Urolithiasis		*P*-value
		Non (n = 853)	Yes (n = 102)	
Age (year)	66 ± 18	66 ± 18	66 ± 16	0.875^#^
Gender (male)	255 (26.7)	216 (25.3)	39 (38.2)	0.005^¥^
Diabetes mellitus	408 (42.7)	360 (42.2)	48 (47.1)	0.349^¥^
Hypertension	492 (51.5)	432 (50.6)	60 (58.8)	0.118^¥^
Congestive heart failure	42 (4.4)	37 (4.3)	5 (4.9)	0.797^¥^
Coronary artery disease	105 (11.0)	94 (11.0)	11 (10.8)	0.943^¥^
Stroke	205 (21.5)	187 (21.9)	18 (17.6)	0.320^¥^
Prior history of UTI				0.016^¥^
None	641 (67.1)	585 (68.6)	56 (54.9)	
Once	180 (18.8)	155 (18.2)	25 (24.5)	
Twice	75 (7.9)	66 (7.7)	9 (8.8)	
Thrice or more	59 (6.2)	47 (5.5)	12 (11.8)	
Indwelling Foley catheter	62 (6.5)	54 (6.3)	8 (7.8)	0.558^¥^
Afebrile	355 (37.2)	314 (36.8)	41 (40.2)	0.504^¥^
Bacteremia	416 (43.6)	366 (42.9)	50 (49.0)	0.239^¥^
Uroseptic shock	176 (18.4)	150 (17.6)	26 (25.5)	0.052^¥^
Acute kidney injury	105 (11.0)	82 (9.6)	23 (22.5)	< 0.001^¥^
Length of hospital stay (day)	9 ± 5	9 ± 5	9 ± 4	0.861^#^
All-cause in-hospital mortality	6 (0.6)	6 (0.7)	0 (0.0)	0.507^¥^
Hospitalized serum creatinine (mg/dL)	1.47 ± 1.39	1.46 ± 1.39	1.58 ± 1.41	0.392^#^
Baseline eGFR (mL/min/1.73 m^2^)	76.84 ± 30.14	76.89 ± 30.55	76.36 ± 26.61	0.865^#^
BUN/creatinine ratio > 20	305 (31.9)	273 (32)	32 (31.4)	0.897^¥^
Mean white blood cell (10^3^/μL)	13.28 ± 5.94	13.21 ± 5.91	13.82 ± 6.24	0.330^#^
Platelets (10^3^/μL)	205.56 ± 112.65	206.21 ± 115.61	200.12 ± 84.17	0.606^#^
*Escherichia coli*	764 (80.0)	690 (80.9)	74 (72.5)	0.047^¥^
*Proteus* species	22 (2.3)	14 (1.6)	8 (7.8)	0.001^¥^
*Klebsiella* species	67 (7.0)	60 (7.0)	7 (6.9)	0.949^¥^
*Enterococcus* species	32 (3.4)	30 (3.5)	2 (2.0)	0.567^¥^
*Pseudomonas* species	48 (5.0)	43 (5.0)	5 (4.9)	0.952^¥^
Nephrotoxic agents	571 (60.0)	514 (60.5)	57 (55.9)	0.371^¥^
Cephalosporins	658 (68.9)	584 (68.5)	74 (72.5)	0.400^¥^
Fluoroquinolones	97 (10.2)	85 (10)	12 (11.8)	0.570^¥^
Penicillins	112 (11.7)	99 (11.6)	13 (12.7)	0.735^¥^
Aminoglycosides	70 (7.3)	64 (7.5)	6 (5.9)	0.553^¥^
Carbapenems	55 (5.8)	52 (6.1)	3 (2.9)	0.196^¥^
Multidrug-resistance bacterial isolate	327 (34.2)	290 (34.0)	37 (36.3)	0.647^¥^

Data are expressed as mean ± SD or number (percentage) ^#^Student T-test; ^¥^Chi-Square test or Fisher’s exact test.

*UTI* urinary tract infection, *eGFR* estimated glomerular filtration rate, *BUN* blood urea nitrogen.

**Table 5:** Univariate and multivariate logistic regression analyses of factors related to acute kidney injury in hospitalized urinary tract infection patients without hydronephrosis.

Covariate	Univariate			Multivariate		
	*β*	OR (95% CI)	*P*-value	*β*	OR (95% CI)	*P*-value
Age (year)	0.029	1.029 (1.015–1.043)	< 0.001	0.008	1.008 (0.990–1.027)	0.371
Gender (male)	0.260	1.297 (0.835–2.012)	0.247			
Diabetes mellitus	0.437	1.549 (1.031–2.326)	0.035	0.126	1.134 (0.715–1.799)	0.592
Hypertension	0.954	2.596 (1.665–4.048)	< 0.001	0.754	2.125 (1.255–3.599)	0.005
Congestive heart failure	0.990	2.691 (1.282–5.646)	0.009	0.266	1.305 (0.539–3.161)	0.555
Coronary artery disease	0.514	1.673 (0.952–2.941)	0.074			
Stroke	0.210	1.234 (0.769–1.980)	0.384			
Indwelling Foley catheter	0.480	1.615 (0.795–3.284)	0.185			
Afebrile	0.442	1.556 (1.034–2.340)	0.034	0.282	1.326 (0.810–2.169)	0.262
Bacteremia	0.528	1.696 (1.128–2.550)	0.011	0.460	1.585 (0.986–2.548)	0.057
Uroseptic shock	1.463	4.318 (2.814–6.627)	< 0.001	1.544	4.683 (2.880–7.613)	< 0.001
Urolithiasis	1.007	2.737 (1.632–4.591)	< 0.001	0.897	2.451 (1.369–4.389)	0.003
Baseline eGFR (mL/min/1.73 m^2^)	− 0.018	0.982 (0.976–0.989)	< 0.001	− 0.013	0.987 (0.979–0.996)	0.004
BUN/creatinine ratio > 20	0.306	1.358 (0.893–2.067)	0.153			
Mean white blood cell (10^3^/μL)	0.058	1.059 (1.027–1.092)	< 0.001	0.067	1.069 (1.034–1.105)	< 0.001
Platelets (10^3^/μL)	− 0.002	0.998 (0.996–1.001)	0.151			
Nephrotoxic agents	− 0.806	0.447 (0.296–0.675)	< 0.001	− 0.673	0.510 (0.319–0.816)	0.005
Multidrug-resistance bacterial isolate	0.279	1.322 (0.872–2.003)	0.188			

Cox & Snell R^2^ = 0.119, Nagelkerke R^2^ = 0.240.

*eGFR* estimated glomerular filtration rate, *BUN* blood urea nitrogen.

## Discussion

UTI is a common complication in patients with urolithiasis. Urolithiasis is a common cause of urinary tract obstruction predisposing to UTI^9^. A previous study showed that urinary obstruction is a predictor of the development of septic shock in UTI patients^10^. In this study, we identified that the presence of ureteral stone with hydronephrosis is a risk factor for AKI in UTI patients with urolithiasis. In addition, urolithiasis is a risk factor for AKI in UTI patients without hydronephrosis. To our knowledge, this is the first study to demonstrate that urolithiasis without hydronephrosis is associated with an increased risk for AKI in patients with UTI.

Urolithiasis is a major cause of obstructive pyelonephritis, accounting for 65% of obstructive pyelonephritis events^11^. Urinary stasis provides the time and opportunity for bacteria to adhere to the urothelium, multiply, and infect the host^12^. In addition, stones moving down the ureter result in inflamed narrowing of the ureter or injuries, which could easily cause infection^13^. Obstructive uropathy is not a rare cause of UTI. A previous study showed that the rate of obstructive uropathy in patients clinically diagnosed with a simple pyelonephritis was approximately 6%^14^. Obstructive UTI is associated with a high recurrence rate. In a long-term follow-up study in patients with obstructive pyelonephritis, the incidence of recurrent obstructive pyelonephritis and recurrent UTI was 11% and 33%, respectively^11^. The existence of ureteral stone is the most common cause of urosepsis in patients with urinary tract obstruction^10^. UTI in the presence of urinary tract obstruction is a serious clinical situation. In the case of acute obstructive uropathy, the increased intrarenal pelvic pressure decreases the drug delivery to the kidney^15^. Both urolithiasis and obstructive uropathy have an impact on the severity of urosepsis^16^, the presence of urinary tract obstruction in patients with acute pyelonephritis is associated with an increased risk for urosepsis and uroseptic shock^17,18^. In the current study, the presence of ureteral stone with hydronephrosis was an independent risk factor for AKI development in UTI patients with urolithiasis.

Obstructive nephropathy is the major cause of non-infectious urolithiasis associated AKI^19^. Renal vasoconstriction and inflammation can occur in response to increased intratubular pressure, and cause ischemia injury. If ischemia persists, glomerulosclerosis, tubular atrophy, and interstitial fibrosis occur^20^. In a rat model, complete unilateral obstruction for 24 h resulted in irreversible loss of function in 15% of the nephrons of the affected kidney^21^. Obstructive AKI caused by urolithiasis is not common, accounting for only approximately 1–2% of all AKI events^19^. A previous study reported that the prevalence of AKI among patients with non-infectious ureteral stone was 0.72%^22^. Infection is one of the leading causes of AKI in patients with ureteral stone^23^, and infection with concomitant obstructive uropathy causes loss of function far more rapidly than a simple obstructive uropathy^24^. Yamamoto et al. found that approximately 80% of patients with acute obstructive pyelonephritis induced by upper ureteral stone had elevated serum creatinine levels above the normal range^25^. AKI is associated with an increased risk for mortality in patients with upper ureteral stone with concomitant urosepsis^26^. Untreated infection with concomitant urinary tract obstruction can lead to life-threatening sepsis. After the administration of appropriate antibiotics, interventions, such as drainage for source control, should be performed within 12 h after the diagnosis of sepsis, according to the recommendation from the Surviving Sepsis Campaign 2016^27^. The management of infected hydronephrosis secondary to urolithiasis is prompt decompression of the renal collecting system^28^. Optional methods of decompression include percutaneous nephrostomy and retrograde ureteral stenting^16^. Decompression of the renal collecting system increases the renal plasma flow and delivery of antibiotics to both the renal parenchyma and urine^29^. Moreover, the release of inner pressure from the upper urinary tracts reduces the invasion of bacteria into the bloodstream or renal parenchyma^30^. Both the increase of antibiotic penetration into the affected renal unit and the decrease of bacterial invasion can facilitate infection control. Further, the severity of tubular atrophy and interstitial fibrosis increases if the duration of obstruction is extended^31^, and a delayed relief of ureteral obstruction decreases long-term renal function^32^. If obstruction is relieved early and infection is controlled, the renal parenchyma has a great potential for recovery^23^. Delay or omission of renal decompression will increase the rate of sepsis and mortality^11^. Because UTI patients with concomitant ureteral stone resulting in urinary tract obstruction are predisposed to develop severe complications, such as septic shock or AKI, timely decompression of the renal collecting system through percutaneous nephrostomy or retrograde ureteral stenting after starting empiric antibiotic therapy is recommended for those with a critical status^33^.

Urolithiasis is a source of infection^34^, and UTIs are frequently associated with almost all chemical types of kidney stones^35^. Urolithiasis is the one of most common urological disorders associated with complicated pyelonephritis^36^ and is also a risk factor for treatment failure in patients with acute pyelonephritis^37^. Because the amount of bacteria on the stone surface can increase despite antibiotic therapy, eradication of the associated UTI is only possible after the stone has been completely removed^38^. According to the Nationwide Inpatient Sample survey between 1999 and 2009, the incidence of urolithiasis-associated sepsis and severe sepsis is increasing^39^. Urolithiasis is also a risk factor of septic shock in patients with UTI^7^, and sepsis is one of the most common etiologies of AKI^40^. In the current study, urolithiasis without hydronephrosis was associated with an increased risk for AKI in patients with UTI. Early imaging examination to identify urolithiasis and complete removal of urolithiasis after ameliorating the symptoms of sepsis are crucial for patients with UTI.

Our study had several limitations. First, this was a single-center study with a retrospective design. A multicenter prospective study with a larger sample size will be needed to confirm our results. Second, we did not routinely examine the stone characteristics in this study. Further studies evaluating the chemical types of stones will be needed to confirm their impact on AKI in patients with UTI. Third, nephrotoxic agents were usually avoided in hospitalized patients with impaired baseline renal function or at risk of AKI. Because of the selection bias, the use of nephrotoxic agents was associated with decreased risk of AKI in univariate analysis and/or multivariate analysis. Forth, patients with history of recurrent AKI may be prone to developing AKI in the presence of UTI and urolithiasis. However, this is a retrospective study, data regarding the frequency of AKI episode before the study period was incomplete to determine their impact on the subsequent development of AKI in patients with UTI and urolithiasis. Fifth, serum bicarbonate was examined if patients develop severe AKI. Most of our patients did not experience severe AKI [overall 57 of 1113 patients (5.1%) had AKI stage 3], data of serum bicarbonate was incomplete for analysis of its relation with AKI.

In conclusion, we demonstrated that patients with ureteral stone complicated with hydronephrosis have a higher risk of developing AKI among UTI patients with urolithiasis, and urolithiasis remains a risk factor for AKI among UTI patients without hydronephrosis. Therefore, timely decompression of the renal collecting system for infectious hydronephrosis secondary to urolithiasis and complete stone removal after controlling infection are crucial for UTI patients with urolithiasis.

## Methods

### Patient selection

This retrospective observational study covered the period from January 2006 to April 2019 and was conducted at Chia-Yi Christian Hospital, a tertiary referral center in the southwestern part of Taiwan. All procedures followed were in accordance with the ethical standards of the responsible committee on human experimentation (institutional and national) and with the Helsinki Declaration of 1975, as revised in 2000. This retrospective observational study was performed after obtaining ethical approval from the Institutional Review Board of Chiayi Christian Hospital (approval no. CYCH-IRB-2019061). This retrospective observational study was using de-identified and routine diagnosis/treatment data, thus CYCH-IRB-2019061 approved waiver of informed consent from all patients for being included in the study.

A total of 1113 consecutive hospitalized patients with UTI without any other concurrent infectious disease were enrolled. All enrolled patients fulfilled the following criteria: (a) with UTI symptoms and > 10^5^ colony-forming units/mL of bacterial isolates from a urine specimen; (b) had available antimicrobial susceptibility test results; and (c) underwent complete imaging examination, including ultrasonography, intravenous urography, or computed tomography, and had complete data of the required laboratory tests. Patients aged < 18 years, with concurrent infection other than UTI, without imaging studies, with incomplete data, or undergoing regular dialysis therapy were excluded from this study. In patients with AKI, BUN/creatinine ratio was examined for pre-renal diseases, patients’ drug history was reviewed to find the use of nephrotoxic agents. All patients had received imaging survey to detect anatomical abnormality of urinary tract. Those patients who hydronephrosis was found by sonography study would receive CT scan to identify the etiology of hydronephrosis.

Inpatients were assessed using standard laboratory and diagnostic procedures. For further analysis, we collected clinical data in a standard form, including demographic characteristics (age and sex), comorbidities (coronary artery disease, CHF, stroke, DM, or hypertension), presence of an indwelling Foley catheter, vital signs (blood pressure and ear temperature), laboratory results (WBC count, platelet count, serum creatinine level, and eGFR at baseline and after hospitalization), prior history of UTI, existence of a urinary tract abnormality (urolithiasis or hydronephrosis), length of hospital stay, causative microorganisms (*E. coli*, *Proteus* species, *Klebsiella* species, *Enterococcus* species, and *Pseudomonas* species), antimicrobial resistance pattern, and the use of antibiotics or nephrotoxic agents.

### Definition and examples of clinical covariates

The eGFR was determined using the Chronic Kidney Disease Epidemiology Collaboration creatinine equation and expressed in mL/min/1.73 m^2^^41,42^. AKI was defined as an increase in serum creatinine to ≥ 2.0 times the baseline value according to the Kidney Disease: Improving Global Outcomes Clinical Practice Guideline criteria for serum creatinine value for AKI stages 2 and 3^43^. Hydronephrosis was defined as dilatation of the renal pelvis and calyces in imaging studies. Uroseptic shock was defined as sepsis-induced hypotension (systolic blood pressure [SBP] < 90 mmHg or mean arterial pressure < 70 mmHg or a decrease in SBP by > 40 mmHg lasting for at least 1 h, despite adequate fluid resuscitation and in the absence of other causes of hypotension)^44^. An afebrile status was defined as a single temperature not increasing to > 38.3 °C (101°F)^45^. Nephrotoxic agents were defined as the use of nonsteroidal anti-inflammatory drugs, aminoglycosides, or contrast media. Multiple drug resistance (MDR) was defined as non-susceptibility to at least three antimicrobial categories ^46^.

### Statistical analysis

All analyses were performed using SPSS version 17.0 (SPSS Inc., Chicago, IL, USA). Continuous variables are expressed as mean ± standard deviation, and categorical variables are expressed as number (percentage). Univariate analyses were performed using Student’s t-test and Mann–Whitney *U*-test for continuous variables, and the chi-square test and Fisher’s exact test for categorical variables. Multivariate logistic regression analyses were applied to identify risk factors associated with AKI during hospitalization. Only variables significant at the 0.05 level in univariate logistic regression analysis were selected for the subsequent multivariate analysis. The goodness-of-fit of the logistic regression model was assessed using the Cox & Snell test, and the explanatory power was reported with Nagelkerke’s pseudo-*R*-square. P < 0.05 was considered statistically significant.
